# A device for precision positioning and alignment of room lasers to diminish their contribution to patient setup errors

**DOI:** 10.1120/jacmp.v8i4.2398

**Published:** 2007-10-10

**Authors:** Ivan A. Brezovich, Stephen Jordan

**Affiliations:** ^1^ Department of Radiation Oncology University of Alabama at Birmingham Birmingham Alabama U.S.A.

**Keywords:** radiation therapy, room lasers, alignment, patient positioning

## Abstract

This report presents an analysis of patient setup errors resulting from inaccurately positioned wall lasers. It suggests that laser beams should agree within 0.2 degree or better with the machine axes that they are delineating. For typical simulator and treatment rooms having wall‐to‐isocenter distances of 3 m, this requirement is satisfied when the beam‐emitting aperture is mounted within about 1.0 cm from the intersection of the respective machine axis with the wall. To achieve the required precision, we developed and clinically tested a simple, inexpensive tool, the Laser Placer (LP). The essential component of the LP is a cube with mirror surfaces that is aligned with the machine axes using built‐in spirit levels and the light field and crosshairs of the collimator. Wall, ceiling, and sagittal lasers are installed and aligned according to reflections of their beams by the cube, and reference lines provided by the LP. Measurements showed that, even in new accelerator installations performed by highly experienced technicians, wall lasers are often mounted off target by more than 1.5 cm. Such inaccuracies can contribute systematic errors of 2 mm or more to the random setup errors attributable to interfraction movement in patient anatomy. To keep setup errors to a minimum, medical physicists should check beam orthogonality in addition to beam congruence at isocenter as recommended by the TG‐40 quality assurance protocol from the American Association of Physicists in Medicine.

PACS number: 87.53.‐j

## I. INTRODUCTION

Room‐mounted positioning lights are valuable aids for setting up patients who are undergoing radiotherapy. These lights are used during conventional or computed tomography simulation to place marks on the skin of patients or on their immobilization devices, so that the simulation geometry can be reproduced in the treatment room.

The first positioning lights were similar to slide projectors: they used incandescent light bulbs to project a crosshairs onto the patient. Laser pointers soon replaced the original projectors, because the resulting bright red dots were visible even under ambient light.^(1)^ Boyer^(2)^ described the merits of a beam splitter and cylindrical lenses to generate two laser fan beams that intersect along the machine axes, thereby forming a crosshairs that is easier to align with skin marks than is a single laser dot. Commercial devices now utilize that method or provide two separate diode lasers and lenses to create the fan beams.

Concerning accuracy of lasers, the Radiation Therapy Task Group 40 Report from the American Association of Physicists in Medicine (AAPM)^(3)^ recommends that lasers be tested daily and kept to a tolerance of 2 mm, implying that laser beams need to approach isocenter within that distance. Horwitz and Forsaith^(4)^ pointed out that serious inaccuracies in patient positioning can result if laser beams pass through isocenter without regard to orthogonality to the machine axes. However, those authors did not provide estimates of patient setup errors attributable to deviations from strict orthogonality or acceptable tolerances.

As an aid for obtaining orthogonality, Goitein proposed to mount a glass plate in front of the laser aperture.^(5)^ By rotating the plate, the beam can be shifted in small increments parallel to itself until it coincides with the appropriate accelerator axis. Modern lasers are equipped with mechanical means for precise shifts along two orthogonal axes, and they thereby offer some leeway in the tedious initial mounting of the laser housings. These positioning aids are provided in addition to the usual fine adjustments for tilt and rotation of beams around their principal axes.

Despite the sophistication of modern lasers, installation and alignment are often accomplished using general‐purpose tools such as plumb bobs dropped from the ceiling, long spirit levels, and water‐filled hoses. Such tools may be acceptable for an initial installation, but they are cumbersome for routine quality assurance checks or quick replacement of a defective laser in a busy department.

A tool designed specifically for laser installations in radiotherapy rooms has been suggested by Horwitz and Forsaith.^(4)^ It consists of a cube with mirrors on its sides and a low‐power telescope for alignment with the gantry axis. However, having been designed before fan beam and sagittal lasers were introduced, the device does not provide guidance for rotational alignment of the fan lines, nor for mounting and aligning sagittal lasers. A sophisticated device that incorporates several potentially fragile micrometer drives, the Radac‐2100 (Medtec, Orange City, IA), is commercially available.

In the present paper, we investigate patient setup errors resulting from inaccurately mounted wall lasers and suggest tolerances for their positioning. We also describe a very simple, relatively rugged, in‐house‐developed device, the Laser Placer (LP), that aids in the installation and alignment of lasers, and we report on its clinical performance. The device costs less than $1000 to manufacture and could be duplicated by any good machine shop.

## II. MATERIALS AND METHODS

### A. Estimation of setup errors

**Figure 1:**
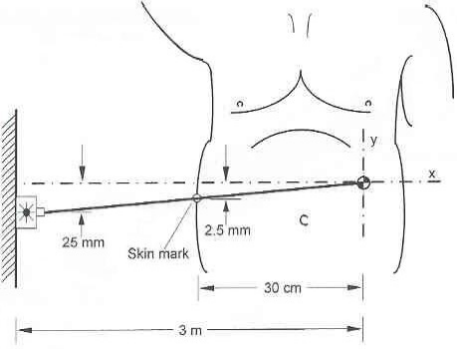
A laser that is inaccurately mounted on the wall causes an error in patient setup, even when the beam is aiming precisely at isocenter (figure not to scale).

Fig. 1 illustrates the potential patient setup error caused by an inaccurately mounted wall laser. We assume that the isocenter of the accelerator (or simulator) is 3 m from the wall, and that the laser aperture is mounted 25 mm from its desired position at the intersection of the transverse machine axis with the wall. The resulting deviation from orthogonality to the gantry axis is ${\text{tan}}^{-1}(25/3000)=0.477$ degree. If a tumor located 10 cm to the patient's left is irradiated, and the patient is 40 cm wide, the skin mark on the patient's right lateral border is located 30 cm from isocenter. Using similar triangles, a patient who has been set up according to an accurately drawn skin mark will clearly be irradiated 2.5 mm superior to the planned position if only this one laser is used for setup. Even in the more likely scenario that the tumor is centrally located, the skin marks are situated 20 cm from isocenter, causing a 1.66 mm error.

If the laser on the opposite wall is also used, rotational and shift errors will result, depending on the particular situation. For example, if the second laser is perfectly positioned, the shift error at isocenter will be 0.8 mm, and the rotational error will be 0.36 degree. To assure that laser‐related setup errors are at or below 1 mm and that rotational errors are avoided, the laser beams must agree with the accelerator axes within 0.19 degree, corresponding to an aperture offset of 1.0 cm or less in a typical treatment room with a wall‐to‐isocenter distance of 3 m.

### B. Principle of operation

**Figure 2:**
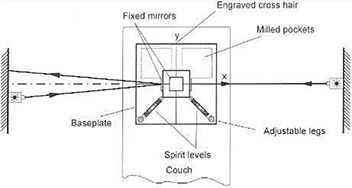
Principle of operation of the in‐house‐designed and tested Laser Placer. The beam of a properly mounted laser is reflected back into itself. The correct position for the aperture of an imprecisely mounted laser lies half way between its original position and the intersection of the reflected beam with the wall.

**Figure 3:**
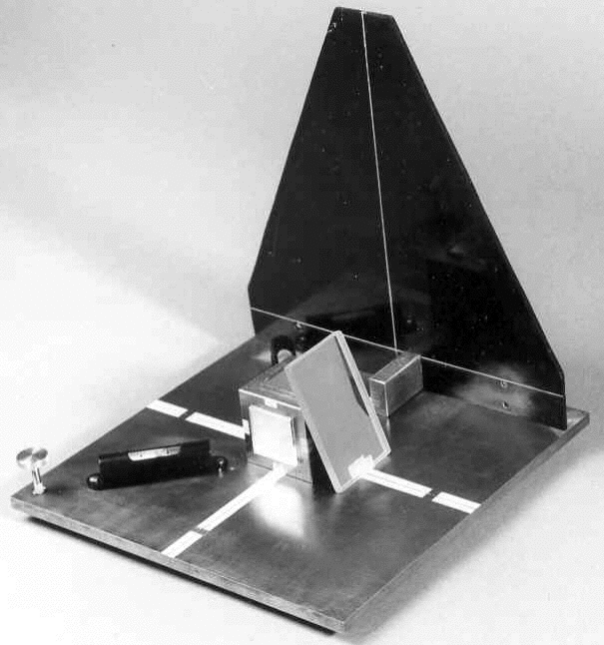
The in‐house‐designed and tested Laser Placer with the portable mirror in place for adjustment of a sagittal laser. The triangular plastic ruler provides vertical and horizontal reference lines for checking and adjusting the rotational alignment of the laser beams.

The LP consists of a precisely machined steel cube centered atop a horizontal base plate (Fig. 2). Lines are engraved on the top surface of the base plate and on the cube to mark their respective centers. Mirrors are attached to the lateral surfaces and to the top surface of the cube. The LP is positioned on the treatment couch so that the base plate is horizontal, the center of the cube coincides with machine isocenter, and the mirrors are perpendicular to the respective beam axes. Beams of properly positioned wall and ceiling lasers intercept the cube at the reference lines marking isocenter and are reflected into themselves (Fig. 3). The reflected light from an inaccurately mounted laser misses the aperture by double the positional error, indicating that the laser aperture should be shifted to a point half way between its original position and the position of the reflected beam. Although surface‐coated mirrors would be preferable, the drawbacks of regular mirrors are negligible. Refraction in glass of 3 mm thickness causes only a slight shift in the position of a reflected beam, and does not affect the angle of reflection. For an accurately positioned laser aperture, the beam is perpendicular to the mirror surface, and therefore angle and position of the reflected beam are not affected by the refraction.

The LP is supported by three pointed legs. One fixed leg is located beneath the center of the cube, and two screw‐adjustable legs are located along diagonals of the base plate near adjacent corners. A spirit level is mounted along each of these (orthogonal) diagonals for horizontal alignment. The bottom of the plate is hollowed in the area opposite the adjustable legs. The ensuing uneven weight reduction shifts the center of gravity into the triangle defined by the three legs, providing a steady stand for the LP. Furthermore, because most of the weight is borne by the central leg, the LP can be rotated about its vertical axis without perturbing the position of the center of the cube.

To estimate the accuracy with which the LP has to be manufactured, we note that the error introduced by imprecision in a measuring tool should be small compared to the error tolerance of the object to be measured. This implies that the faces of the cube have to be orthogonal to one another within substantially less than 0.19 degree—say, 0.02 degree. Such precision can readily be achieved by a good machine shop. The sensitivity of the spirit levels, quoted by the manufacturer (L.S. Starrett Company, Athol, MA) as one division of bubble deflection per 0.017 degree tilt angle, also meets this requirement.

**Figure 4:**
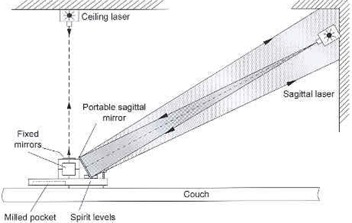
Side view of the in‐house‐designed and tested Laser Placer. The portable mirror has been tilted so that the sagittal beam is reflected back toward the laser aperture.

For rotational adjustment of laser lines, a portable plastic plate with precisely engraved lines is provided (Fig. 3). When properly positioned on the base plate, it furnishes horizontal and vertical reference lines to which the laser lines are matched. The lines engraved on the base plate provide guidance for rotational adjustment of the ceiling laser. For mounting and testing the sagittal laser, a portable mirror is provided. This mirror is placed on the base plate and leaned against the precisely machined upper front edge of the cube. Its tilt angle is then adjusted so that the reflected beam aims toward the laser aperture (Fig. 4). A narrow strip of tape affixed at the center of the bottom edge of the mirror acts as a pivot around which the mirror can freely rotate, thus assuring that its back surface maintains contact along the edge of the cube. Again, accuracy is established when a beam that is directed at isocenter is reflected into itself and matches the vertical line on the plastic plate.

### C. Using the LP

**Figure 5:**
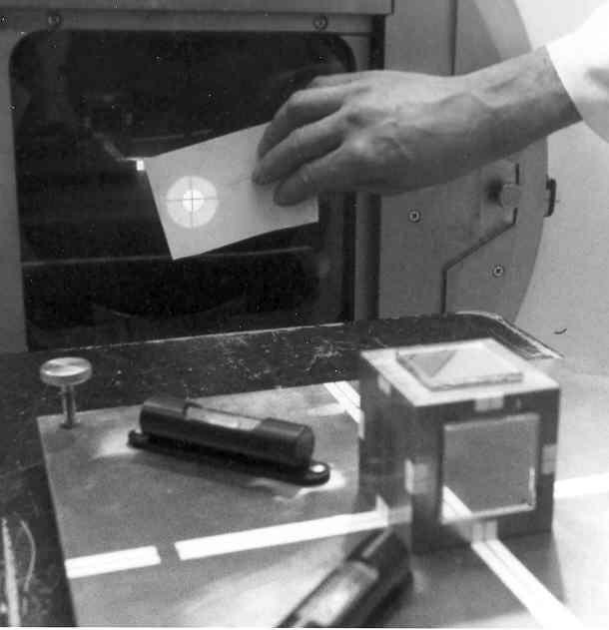
The in‐house‐designed and tested Laser Placer has been accurately aligned with the accelerator axes. The reflected image of the crosshairs, seen in the bright ring of light around the cutout, matches the forward projection of the crosshairs (visible within the cutout).

The LP is placed on the treatment couch, leveled, and positioned at isocenter with the help of the linear couch motions and the optical and mechanical distance indicators of the collimator. With the gantry turned horizontal, the jaws set to about 3 cm by 3 cm and the field light turned on, the LP is rotated about its fixed leg until the reflected image of the collimator crosshairs coincides with its forward projection. An aperture, consisting of a sheet of cardboard with a hole cut in its middle, is held against the collimator face to facilitate this task (Fig. 5). The gantry is then rotated to the opposite lateral position, and the test is repeated. Unless the accelerator is absolutely rigid and perfectly aligned, a slight discrepancy between the original and the reflected images of the crosshairs will be noted. The LP is then rotated to cut the mismatch in half (“split the difference”). Accurate rotational alignment is achieved when the images at both gantry positions are identical. The LP requires no further adjustment until all lasers are installed and aligned.

## III. RESULTS

### A. Precision and accuracy tests

To assess accuracy and repeatability, we used the LP to reposition and align the lasers in one of our treatment rooms. Because we were primarily interested in the performance of the LP, we did not track laser position errors, except for the sagittal laser. The sagittal laser had been about 3 cm off target and was repositioned (as we were able to later deduce from the position of the original mounting holes on the wall). After the alignment procedure, all laser beams intersected precisely at isocenter. Corresponding lines remained matched within better than 1 mm to distances beyond 1 m from isocenter. The vertical lines produced by the wall lasers matched equally well with one another and with the transverse line of the ceiling laser. The line cast by the sagittal laser agreed with the longitudinal line of the ceiling laser to better than 1 mm over the entire length of the couch top. The beams of the two wall lasers intercepted the apertures of their counterparts on the opposite wall within 2 mm.

The tests were repeated with the LP rotated by 180 degrees about its vertical axis. The rationale for this procedure was that any manufacturing defects in the LP (especially angular imprecisions of the cube faces) would manifest themselves as an erroneous indication of poor laser alignment. No such erroneous indications were noted, and all lasers appeared accurately aligned as before.

To test reproducibility, the LP was removed from the couch, and the table top and gantry were moved arbitrarily. When the LP was replaced on the couch and adjusted, the positions of the reflected laser beams on the walls in relation to reference marks placed during the original alignment procedure were recorded. The experiment was repeated 10 times by each of 2 observers. In all experiments, the reflected laser line, 3 mm in width, covered the pencil marks, indicating reproducibility within ±1.5 mm. (The width of the reflected line was the result of a requirement to focus the lasers near isocenter; off‐focus blurring ensued at larger distances.)

### B. Clinical Performance

The clinical practicality of the LP was tested when it was used to position and align replacement lasers in the simulator room. Using the device and the original lasers, the proper position for the new lasers was found. According to a cursory inspection, the wall and ceiling lasers had been positioned within 1.5 cm from the target, but the sagittal laser was more than 3 cm in error. After the new lasers were mounted, fine positional, rotational, and tilt alignments were then made using the internal worm screws provided by the manufacturer (LAP Laser Applications L.C., Boca Raton, FL). The entire procedure took less than 90 minutes, excluding the time required to bolt the lasers to the walls.

The LP was then used to check the lasers in our two remaining treatment rooms. Although all lasers beams agreed with isocenter to better than 1 mm, typical aperture position errors were about 1 cm. However, in one of the rooms, the aperture of the wall laser that produced the vertical line on the patient's right (head first, supine position) was 2.8 cm in error in the caudad direction. The isocenter‐to‐wall distance was 2.5 m. The laser on the opposite wall, which was located 4.3 m from isocenter, was 2.5 cm off target in the same direction. The sagittal laser, mounted 3.6 m from isocenter, was off by 3.2 cm.

Because the positional errors of these three lasers exceeded the range of the internal adjustment mechanisms, the lasers had to be removed from their respective walls and remounted. The apertures of the ceiling lasers producing the transverse and parallel lines were off target by 2.0 and 1.5 cm respectively. Laser lines typically required 0.5 degree of rotation, equivalent to about 1 mm discrepancy at 10 cm from isocenter. Using similar triangles, it can be shown that a patient 40 cm in width with a centrally located tumor who has been accurately simulated will be positioned 1.7 mm caudad to the planned position, and rotated by 0.16 degree if set up by matching the lateral skin marks with the two (inaccurately placed) wall lasers. The absence of such large errors in the other treatment room was probably the result of a previous laser alignment using an earlier version of the LP.

After all lasers in the department had been carefully aligned, therapists reported a substantial drop in the number of unsatisfactory patient setups, defined as 5 mm or more disagreement in bony anatomy between simulator and check film. The improvement was most pronounced in the treatment room in which the lasers had been misaligned by more than 2 cm. Therapists reported that, before the alignment was done, about one half of the first‐treatment‐day setups required a shift to meet the ±5 mm match with the simulator films, and a redrawing of the skin marks. Some of the experienced therapists intentionally offset patients during initial setup by one line width (about 2 mm), hoping that the ensuing correction would lead to a quicker match with the simulator film. After alignment of all lasers with the LP, precise setup in the treatment room according to the skin marks placed during simulation resulted in acceptable verification films in more than 80% of the patients.

Table 1 summarizes the positional errors of laser apertures measured with the LP at a nearby community cancer center that is equipped with a simulator and two accelerators. Most of the lasers were accurately positioned. However, some of the devices, especially the ceiling laser in treatment room 1, did not meet the precision suggested by our analysis. In another test, a medical physicist used the LP during acceptance testing and commissioning of a new cancer center. He found the device very practical and easy to use, and commented that he was not aware of any other apparatus that provided the precise guidance offered by the LP for positioning of the sagittal lasers (Rice JR, personal communication).

**Table 1:** Deviation of laser apertures from their correct positions on the wall

Room	Laser type	Location	Wall‐to‐isocenter distance (m)	Deviation (cm) Parallel^a^	Transverse^b^
Simulator	He–Ne gas	Left wall	2.5	0.5	0.1
		Right wall	2.5	0.1	0.1
		Ceiling	3.0	N/A^c^	N/A^c^
		Sagittal	4.0	0.1	N/A
Accelerator 1	He–Ne gas	Left wall	2.6	1.0	0.75
		Right wall	2.6	0.6	1.0
		Ceiling	3.0	1.75	3.0
		Sagittal	4.1	1.5	N/A
Accelerator 2	Solid state	Left wall	3.6	1.5	0.5
		Right wall	3.6	1.0	0.15
		Ceiling	3.0	0.15	0.15
		Sagittal	3.7	0.5	N/A

a Line parallel to the gantry axis—for example, the horizontal line of a wall laser.

b Line perpendicular to the gantry axis—for example, vertical line of a wall laser.

c Laser not working.

## IV. DISCUSSION

Steady improvements in radiation medicine, especially conformal and intensity‐modulated treatments, are placing ever stiffer demands on precise patient setup. Industry has responded by developing better immobilization devices, such as patient‐specific cradles and mouth molds that greatly reduce patient‐induced errors. With the main error source greatly reduced, the need for reducing other error sources has risen.

The authors of the AAPM TG‐40 quality assurance protocol apparently recognized the difficulty of maintaining laser beams aimed precisely at isocenter, and they allowed a rather generous 2‐mm tolerance. Drifts of the laser line resulting from temperature changes, vibrations, and other factors would make it impractical to maintain tighter tolerances, especially for gas lasers. Nevertheless, a 2‐mm error in the treatment room plus a similar one in the simulator room could nearly exhaust a tight safety margin between a tumor and a nearby critical structure.

As this analysis has shown, inaccurately placed laser apertures can contribute an additional 2 mm or more to the total error. Such problems may be encountered even in new machines installed by experienced technicians. However, they can be virtually eliminated by a conscientious medical physicist equipped with the LP. Because the position of a laser does not change unless it is removed from the wall for replacement, another treatment machine is installed, or major room renovations occur, the positioning is a one‐time effort. We therefore recommend that the contribution of laser positioning to patient error be maintained at or below 0.5 mm, corresponding to an angular beam misalignment of 0.1 degree or less, or to 0.5 cm laser position inaccuracy in a typical treatment room with a 3‐m isocenter‐to‐wall distance.

The LP also showed that laser lines are often inaccurately aligned around their main axes. Although rotational misalignments of this kind do not affect the ability to delineate machine axes, they can lead to errors if the lines are used off central ray. The need to use laser lines at a distance from their intersections may arise if the intersection lies on a bolus or on an otherwise unstable surface. Furthermore, laser beams in the sagittal plane are often used as a guide during initial setup for positioning the entire patient or the immobilization device. Again, the LP proved practical for rotational alignment of all laser lines so that they accurately agreed with the respective principal planes along the entire length and width of the couch. The smaller number of unsatisfactory port films reported by our therapists after laser alignment with the LP was a welcome benefit. Considering that none of the errors attributable to inaccurate placement of individual lasers could have caused the high number of poor initial verification films, it is likely that, before all lasers were repositioned and accurately aligned, the cumulative error caused by laser errors in both the simulator room and the treatment vault resulted in frequent violations of the ±5−mm threshold. We admit that this observation was anecdotal and that only a randomized prospective study could have unequivocally established a causal relationship.

Clinical physicists at other institutions probably have devised simpler and cheaper methods and devices for checking laser alignment, including cubes with orthogonal markings and long spirit levels for checking horizontal laser lines. However, while such devices may be equally useful for verifying that lasers are correctly mounted and aligned, they may not be as practical as the LP for finding the correct mounting positions.

## V. CONCLUSION

Inaccurate installation of lasers can contribute substantially to overall setup error of patients. Accuracy cannot be taken for granted, even in installations performed by experienced technicians. By using the device described in the present paper, positioning and alignment errors of lasers are readily detected and, if necessary, corrected. The potential improvement in setup accuracy is a high return for the one‐time investment in effort to correct positional errors. The LP is equally valuable for routine tilt and rotational alignment of laser lines.

## ACKNOWLEDGMENTS

The authors extend their gratitude to Mr. Jerry Sewell for making the Laser Placer (LP) and to Dr. J. Robin Rice for independent testing of the LP and a critical review of the manuscript.

## DISCLOSURE

This work was presented at the 18th Annual Meeting of the American College of Medical Physics, Hershey, Pennsylvania, June 2 – 7, 2001.

## Supporting information

Supplementary Material Files
