# Supplementary material for: A device for precision positioning and alignment of room lasers to diminish their contribution to patient setup errors
Source: J Appl Clin Med Phys. 2007 Oct 10;8(4):45–53. doi: 10.1120/jacmp.v8i4.2398 (PMC5722625; doi:10.1120/jacmp.v8i4.2398)
Supplement: Supplementary file 1 — Supplementary Material Files [file ACM2-8-045-s001.rtf]

Patient set-up errors due to imprecisely mounted lasers


Ivan A. Brezovich, Ph.D., Stephen Jordan

Department of Radiation Oncology

University of Alabama at Birmingham

1824 South 6th Avenue, Birmingham, AL 35294

ibrezovich@uabmc.edu


Corresponding Author:
Ivan A. Brezovich, Ph.D.
Tel: 	(205) 934-4763
FAX:	(205) 975-6161
e-mail:	ibrezovich@uabmc.edu
Presented at the 18th Annual Meeting of the American College of Medical Physics, Hershey, PA, June 2-7, 2001

A device for precision positioning and alignment of room lasers to prevent patient setup errors 


A device for precision positioning and alignment of room lasers to prevent patient setup errors 


Abstract:  An analysis of patient setup errors resulting from inaccurately positioned wall lasers is presented. It suggests that laser beams should agree within 0.2° or better with the machine axes they are delineating. For typical simulator and treatment rooms, having wall-to-isocenter distances of 3 m, this requirement is satisfied when the beam emitting aperture is mounted within about 1.0 cm from the intersection of the respective machine axis with the wall.  To achieve the required precision, we have developed and clinically tested a simple, inexpensive tool, the Laser Placer (LP).  The essential component of the LP is a cube with mirror surfaces that is aligned with the machine axes using built-in spirit levels and the light field and cross hair of the collimator.  Wall, ceiling and sagittal lasers are installed and aligned according to reflections of their beams by the cube, and reference lines provided by the LP.  Measurements showed that even in new accelerator installations performed by highly experienced technicians, wall lasers, are often mounted off target by more than 1.5 cm. Such inaccuracies can contribute systematic errors of two mm or more to the random set-up errors due to moving anatomy. To keep set-up errors to a minimum, medical physicists should check beam orthogonality in addition to beam congruence at isocenter as recommended by the AAPM TG 40 Quality Assurance Protocol. 

Key words:  radiation therapy, room lasers, alignment, patient positioning
PACS number: 87.53.Xd

I. INTRODUCTION	
Room mounted positioning lights are valuable aids for setting up patients undergoing radiotherapy.  These lights are used during conventional or CT simulation to place marks on the skin of patients or on their immobilization devices, so that the simulation geometry can be reproduced in the treatment room.    
The first positioning lights were similar to slide projectors, using incandescent light bulbs to project a cross hair onto the patient.  Laser pointers soon replaced the original projectors, since the bright red dots were visible even under ambient light1.  Boyer2 described the merits of a beam splitter and cylindrical lenses to generate two laser fan beams that intersect along the machine axes, thereby forming a cross hair that is easier to align with skin marks than a single laser dot.  Commercial devices utilize that method, or provide two separate diode lasers and lenses to create the fan beams.  
Concerning accuracy of lasers, the AAPM Radiation Therapy Task Group 40 Report3 recommends that these be tested daily and kept to a tolerance of 2 mm, implying that laser beams need to approach isocenter within that distance.  Horwitz and Forsaith pointed out that serious inaccuracies in patient positioning can result if laser beams pass through isocenter without regard to orthogonality to the machine axes4.  However, the authors do not provide estimates of patient setup errors due to deviations from strict orthogonality or acceptable tolerances.  
As an aid for obtaining orthogonality, Goitein proposed to mount a glass plate in front of the laser aperture5.  By rotating the plate the beam can be shifted in small increments parallel to itself until it coincides with the appropriate accelerator axis.  Modern lasers are equipped with mechanical means for precise shifts along two orthogonal axes, and thereby offer some leeway in the tedious initial mounting of the laser housings.  These positioning aids are provided in addition to the usual fine adjustments for tilt and rotation of beams around their principal axes.
Despite the sophistication of modern lasers, installation and alignment is often done using general purpose tools like plumb bobs dropped from the ceiling, long spirit levels and water filled hoses.  While such tools may be acceptable for an initial installation, these are cumbersome for routine quality assurance checks or quick replacement of a defective laser in a busy department.  A tool designed specifically for laser installations in radiotherapy rooms has been suggested by Horwitz and Forsaith4.  It consists of a cube with mirrors on its sides and a low power telescope for alignment with the gantry axis.  However, having been designed before fan beam and sagittal lasers were introduced, the device does not provide guidance for rotational alignment of the fan lines, or for mounting and aligning sagittal lasers.  A sophisticated device that incorporates several potentially fragile micrometer drives, RADAC-2100™ (MED-TEC Inc. Orange City, IA), is commercially available.  
In this paper we investigate patient setup errors resulting from inaccurately mounted wall lasers and suggest tolerances for their positioning.  We also describe a very simple, relatively rugged in-house developed device, the Laser Placer (LP), for aiding the installation and alignment of lasers, and report on its clinical performance.  The device costs less than $1000 to manufacture and could be duplicated by any good machine shop.
II. METHODS AND MATERIALS
A. Estimation of setup errors
Figure 1 illustrates the potential patient setup error caused by an inaccurately mounted wall laser. We assume that the isocenter of the accelerator (or simulator) is 3 m (10') from the wall, and that the laser aperture is mounted 25 mm (1") from its desired position at the intersection of the transverse machine axis with the wall.  The resulting deviation from orthogonality to the gantry axis is tan-1(25/3000) = 0.477°.  If a tumor located 10 cm to the patient's left is irradiated, and the patient is 40 cm wide, the skin mark on the patient's right lateral border is located 30 cm from isocenter.  Using similar triangles, on can see that a patient who has been set up according to an accurately drawn skin mark will be irradiated 2.5 mm superior to the planned position if only this one laser is used for setup.  Even in the more likely scenario that the tumor is centrally located, the skin marks are situated 20 cm from isocenter, causing a 1.66 mm error.  If the laser on the opposite wall is also used, rotational and shift errors will result, depending on the particular situation.  For example, if the second laser is perfectly positioned, the shift error at isocenter will be 0.8 mm and the rotational error 0.36º.  To assure that laser-related setup errors are at or below 1 mm and rotational errors are avoided, the laser beams must agree with the accelerator axes within 0.19º, corresponding to an aperture offset ≤ 1.0 cm in a typical treatment room with 3 m wall-to-isocenter distance.
B.  Principle of Operation
The LP consists of a precisely machined steel cube centered on top of a horizontal base plate (Fig. 2).  Lines are engraved on the top surface of the base plate and on the cube to mark their respective centers.  Mirrors are attached to the lateral surfaces and to the top surface of the cube.  The LP is positioned on the treatment couch so that the base plate is horizontal, the center of the cube coincides with machine isocenter, and the mirrors are perpendicular to the respective beam axes.  Beams of properly positioned wall and ceiling lasers intercept the cube at the reference lines marking isocenter, and are reflected into themselves (Fig. 3).  The reflected light from an inaccurately mounted laser misses the aperture by double the positional error, indicating that the laser aperture should be shifted to a point half way between its original position and the position of the reflected beam.  Although surface-coated mirrors would be preferable, the drawbacks of regular mirrors are negligible.  Refraction in the 3 mm thick glass causes only a slight shift of the position of a reflected beam, but does not affect the angle of reflection.  For an accurately positioned laser aperture the beam is perpendicular to the mirror surface, and therefore angle and position of the reflected beam are not affected by the refraction.  
The LP is supported by 3 pointed legs.  One fixed leg is located beneath the center of the cube, whereas two screw adjustable legs are located along diagonals of the base plate near adjacent corners.  A spirit level is mounted along each of these (orthogonal) diagonals for horizontal alignment.  The bottom of the plate is hollowed in the area opposite to the adjustable legs.  The ensuing uneven weight reduction shifts the center of gravity into the triangle defined by the 3 legs, providing a steady stand of the LP.  Furthermore, since most of the weight is born by the central leg, one can rotate the LP about its vertical axis without perturbing the position of the center of the cube.    
To estimate the accuracy with which the LP has to be manufactured, we note that the error introduced by imprecision in a measuring tool should be small compared to the error tolerance of the object to be measured.  This implies that the faces of the cube have to be orthogonal to one another within <<0.19°, say 0.02°.  Such precision can be readily achieved by a good machine shop.  The sensitivity of the spirit levels, quoted by the manufacturer (L. S. Starrett Company, Athol, MA) as one division of bubble deflection per 0.017° tilt angle (0.0035" per foot), also meets this requirement.  
For rotational adjustment of laser lines, a portable plastic plate with precisely engraved lines is provided (Fig. 3).  When properly positioned on the base plate, it furnishes horizontal and vertical reference lines to which the laser lines are matched.  The lines engraved on the base plate provide guidance for rotational adjustment of the ceiling laser.  For mounting and testing the sagittal laser, a portable mirror is provided.  This mirror is placed on the base plate, leaned against the precisely machined upper front edge of the cube, and its tilt angle adjusted so that the reflected beam aims toward the laser aperture (Fig 4).  A narrow strip of tape affixed at the center of the bottom edge of the mirror acts as a pivot around which the mirror can freely rotate, thus assuring that its back surface maintains contact along the edge of the cube.  Again, accuracy is established when a beam that is directed at isocenter is reflected into itself and matches the vertical line on the plastic plate.
C.  Using the Laser Placer 
The LP is placed on the treatment couch, leveled, and positioned at isocenter with the help of the linear couch motions and the optical and mechanical distance indicators of the collimator.  With the gantry turned horizontal, the jaws set to about 3x3cm2 and the field light turned on, the LP is rotated about its fixed leg until the reflected image of the collimator cross hair coincides with its forward projection.  An aperture, consisting of a sheet of cardboard with a hole cut in its middle and held against the collimator face, facilitates this task (Fig. 5).  The gantry is then rotated to the opposite lateral position, and the test is repeated.  Unless the accelerator is absolutely rigid and perfectly aligned, there will be a slight discrepancy between the original and the reflected images of the cross hair.  The LP is then rotated to cut the mismatch in half (“split the difference”).  Accurate rotational alignment is achieved when the images at both gantry positions are identical.  The LP requires no further adjustment until all lasers are installed and aligned.  
III. RESULTS
A.  Precision and accuracy tests
To assess accuracy and repeatability, we used the LP to reposition and align the lasers in one of our treatment rooms.  Since we were primarily interested in the performance of the LP, we did not keep track of laser position errors except for the sagittal laser.  The sagittal laser had been about 3 cm off target and repositioned, as we were able to later deduct from the position of the original mounting holes on the wall.  After the alignment procedure, all laser beams intersected precisely at isocenter.  Corresponding lines remained matched within better than 1 mm to distances beyond 1 m from isocenter.  The vertical lines produced by the wall lasers matched equally well one another and the transverse line of the ceiling laser.  The line cast by the sagittal laser agreed with the longitudinal line of the ceiling laser to better than 1 mm over the entire length of the couch top. The beams of the two wall lasers intercepted the apertures of their counterparts on the opposite wall within 2 mm. 
The tests were repeated with the LP rotated by180° about its vertical axis.  The rationale for the procedure was that any manufacturing defects in the LP, especially angular imprecisions of the cube faces, would manifest themselves as an erroneous indication of poor laser alignment.  No such erroneous indications were noted, all lasers appeared accurately aligned as before.
To test reproducibility, the LP was removed from the couch, and table top and gantry were moved arbitrarily.  When the LP was put back on the couch and adjusted, the position of the reflected laser beams on the walls in relation to reference marks placed during the original alignment procedure was recorded.  The experiment was repeated 10 times by each of 2 observers.  In all experiments, the reflected 3 mm wide laser line covered the pencil marks, indicating reproducibility within ±1.5 mm.  (The width of the reflected line was due to the requirement of focusing the lasers near isocenter and the ensuing off-focus blurring at larger distances).  
B.  Clinical Performance
The clinical practicality of the LP was tested when it was used to position and align replacement lasers in the simulator room.  Using the device and the original lasers, the proper position for the new lasers was found.  According to a cursory inspection, the wall and ceiling lasers had been positioned within 1.5 cm from the target, whereas the sagittal laser was more than 3 cm in error.  After the new lasers were mounted, fine positional, rotational and tilt alignments were then made using the internal worm screws provided by the manufacturer (LAP Laser Applications L.C., Boca Raton, FL).  The entire procedure took less than 1.5 hours, excluding the time it took to bolt the lasers to the walls. 
The LP was then used to check the lasers in our two remaining treatment rooms.  While all lasers beams agreed with isocenter to better than 1 mm, typical aperture position errors were about 1 cm.  However, in one of the rooms, the aperture of the wall laser that produced the vertical line on the patient's right (head first, supine position) was 2.8 cm in error in the caudad direction.  The isocenter-to-wall distance was 2.5 m.  The laser on the opposite wall, which was located 4.3 m from isocenter, was 2.5 cm off target in the same direction. The sagittal laser, mounted 3.6 m from isocenter, was off by 3.2 cm.  Since the position errors of these three lasers exceeded the range of the internal adjustment mechanisms, they had to be removed from their respective walls and remounted.  The apertures of the ceiling lasers producing the transverse and the parallel lines were off target by 2.0 and 1.5 cm, respectively.  Laser lines typically required 0.5° of rotation, equivalent to about 1 mm discrepancy at 10 cm from isocenter.   Using similar triangles, one can show that a 40 cm wide patient with a centrally located tumor who has been accurately simulated will be positioned 1.7 mm caudad to the planned position, and rotated by 0.16º if he is set up by matching the lateral skin marks with the two (inaccurately placed) wall lasers.  The absence of such large errors in the other treatment room was probably the result of a previous laser alignment using an earlier version of the LP.
After all lasers in the department had been carefully aligned, therapists reported a substantial drop in the number of unsatisfactory patient setups, defined as ≥5 mm disagreement in bony anatomy between simulator and check film.  The improvement was most pronounced in the treatment room where the lasers had been misaligned by more than 2 cm.  Therapists reported that, before the alignment was done, about one-half of the first-treatment day setups required a shift to meet the ± 5 mm match with the simulator films, and a redrawing of the skin marks.   Some of the experienced therapists intentionally offset patients during initial setup by one line width (about 2 mm), hoping that the ensuing correction would lead to a quicker match with the simulator film.  After alignment of all lasers with the LP, precise setup in the treatment room according to the skin marks placed during simulation resulted in acceptable verification films in more than 80% of the patients.
Table 1 summarizes the positional errors of laser apertures measured with the LP at a nearby community cancer center that was equipped with a simulator and two accelerators.  The majority of lasers were accurately positioned.  However, some of the devices, especially the ceiling laser in treatment room #1, did not meet the precision suggested by our analysis.  In another test, a medical physicist used the LP during acceptance testing and commissioning of a new cancer center.  He found the device very practical and easy to use, and commented that he was not aware of any other apparatus that provided the precise guidance offered by the LP for positioning of the sagittal lasers (J. Robin Rice, PhD, private communication).  
IV. DISCUSSION
Steady improvements in radiation medicine, especially conformal and IMRT treatments, are placing ever stiffer demands on precise patient setup.  Industry has responded by developing better immobilization devices, like patient specific cradles and mouthmolds that greatly reduce patient-induced errors.  With the main error source greatly reduced, the need for reducing other error sources has risen.  
The authors of the AAPM TG 40 QA protocol apparently recognized the difficulty of maintaining laser beams aimed precisely at isocenter, and allow a rather generous 2 mm tolerance.  Drifts of the laser line due to temperature changes, vibrations and other factors would make it impractical to maintain tighter tolerances, especially for gas lasers.  Nevertheless, a 2 mm error in the treatment room plus a similar one in the simulator room could nearly exhaust a tight safety margin between a tumor and a nearby critical structure.  
As this analysis has shown, inaccurately placed laser apertures can contribute an additional two mm or more to the total error.  Such problems may be encountered even in new machines installed by experienced technicians.  However, they can be virtually eliminated by a conscientious medical physicist equipped with the LP.  Since the position of a laser does not change unless it is removed from the wall for replacement, installation of another treatment machine, or major room renovations, the positioning is a one-time effort.  We therefore recommend that the contribution of laser position to patient error be maintained at or below 0.5 mm, corresponding to an angular beam misalignment of ≤ 0.1º or 0.5 cm laser position inaccuracy in a typical treatment room with 3 m isocenter-to-wall distance.  
The LP also showed that laser lines are often inaccurately aligned around their main axes.  While such rotational misalignments do not affect their ability to delineate the machine axes, they can lead to errors if the lines are used off central ray.  The need to use laser lines at a distance from their intersections may arise if the intersection lies on a bolus or on an otherwise unstable surface.  Furthermore, laser beams in the sagittal plane are often used as a guide during initial setup for positioning the entire patient or the immobilization device.  Again, the LP proved practical for rotational alignment of all laser lines so that they accurately agreed with the respective principal planes along the entire length and width of the couch.  The smaller number of unsatisfactory port films reported by our therapists after laser alignment with the LP was a welcome benefit.  Considering that none of the errors due to inaccurate placement of individual lasers could have caused the high number of poor initial verification films, it is likely that the cumulative error caused by laser errors in the simulator room plus those in the treatment vault resulted in the frequent violations of the ± 5 mm threshold before all lasers were repositioned and accurately aligned.  We admit that this observation was anecdotal and that only a randomized prospective study could have unequivocally established a causal relationship.  
Clinical physicists at other institutions probably have devised simpler and cheaper methods and devices for checking laser alignment, including cubes with orthogonal markings and long spirit levels for checking horizontal laser lines.  However, while these may be equally useful for verifying that lasers are correctly mounted and aligned, they may not be as practical as the LP for finding the correct mounting positions.
  
 V. CONCLUSION
Inaccurate installation of lasers can contribute substantially to the overall setup error of patients. Accuracy cannot be taken for granted, even in installations performed by experienced technicians.  Using the device described in this paper, positioning and alignment errors of lasers are readily detected and if necessary corrected.  The potential improvement in setup accuracy is a high return for the one-time investment in effort to correct positional errors.  The LP is equally valuable for routine tilt and rotational alignment of laser lines.

Acknowledgments
The authors extend their gratitude to Mr. Jerry Sewell for making the Laser Placer, and to Dr. J. Robin Rice for the independent testing of the LP and a critical review of the manuscript.

REFERENCES

1.	 Galkin BM, Vogel HB, McArdle GH, Baust GF, Boon R, O'Donnell V. Laser modification to an x-ray collimator: An aid in positioning patients for neurosurgical and radiographic procedures.  Med. Phys. 1975;2(2):79-81.
2.	Boyer AL. Laser 'cross-hair' sidelight. Med. Phys. 1978;5(1):58-60.
3.	Kutcher GJ,   Coia L, Gillin M, et al. Comprehensive QA for radiation oncology: Report of AAPM Radiation Therapy Committee Task Group 40. Med. Phys. 1994;21(4):581-618.
4.	Horwitz NH, Forsaith AL. An instrument for aligning patient-positioning lasers. Med. Phys. 1978;5(2),164-166.
5.	Goitin M.  Mechanism to facilitate the fine adjustment of sidelights.  Med. Phys. 1975;2(4):219-220.


FIGURE CAPTIONS
Fig. 1.	A laser that is inaccurately mounted on the wall causes an error in patient setup, even when the beam is aiming precisely at isocenter. (Figure not to scale.)
Fig. 2.	Principle of operation of the LP.   The beam of a properly mounted laser is reflected back into itself.  The correct position for the aperture for an imprecisely mounted laser lies half way between its original position and the intersection of the reflected beam with the wall. 
Fig. 3.	LP with the portable mirror in place for adjustment of the sagittal laser.  The triangular plastic ruler provides vertical and horizontal reference lines for checking and adjusting the rotational alignment of the laser beams.
Fig. 4.	Side view of the LP.  The portable mirror has been tilted so that the beam of the sagittal beam is reflected toward the laser aperture.
Fig. 5.	The LP has been accurately aligned with the accelerator axes.  The reflected image of the cross hair, seen in the bright ring of light around the cutout matches the forward projection of the cross hair (visible within the cutout).   


Table 1. Deviation of laser apertures from their correct positions on the wall.  
			Wall to isoctr.
distance (m)	Deviation (cm)	
Room	Laser type	Location		Parallel	Transverse	
Simulator	He-Ne gas	lt wall	2.5	0.5	0.1	
		rt wall	2.5	0.1	0.1	
		ceiling	3.0	n/a*	n/a*	
		sagittal	4.0	0.1	n/a	
Accelerator #1	He-Ne gas	lt wall	2.6	1.0	0.75	
		rt wall	2.6	0.6	1.0	
		ceiling	3.0	1.75	3.0	
		sagittal	4.1	1.5	n/a	
Accelerator #2	solid state	lt wall	3.6	1.5	0.5	
		rt wall	3.6	1.0	0.15	
		ceiling	3.0	0.15	0.15	
		sagittal	3.7	0.5	n/a	

Parallel = line parallel to the gantry axis (e.g. the horizontal line of a wall laser), Transverse = line perpendicular to gantry axis (e.g. vertical line of a wall laser), n/a* = laser not working.
